# Knowledge and perceived competence with sexual and gender minority healthcare topics among medical students and medical school faculty

**DOI:** 10.1186/s12909-023-04849-2

**Published:** 2023-12-08

**Authors:** Allison Rhodes, Zachary Barbati, David Tybor, Joshua St. Louis

**Affiliations:** 1 Department of Psychiatry, Tufts Medical Center, Boston, MA USA; 2 Departments of Pathology and Laboratory Medicine, University of California – San Francisco, San Francisco, CA USA; 3 Department of Public Health and Community Medicine, Tufts University School of Medicine, Boston, MA USA; 4 Department of Family Medicine, Tufts University School of Medicine, core faculty, Lawrence Family Medicine residency, Lawrence, MA USA

**Keywords:** LGBTQ, Sexual and gender minority, Faculty development, Medical school education, Perceived competence, Caring for minority populations

## Abstract

**Background:**

Despite changes in social attitudes in the United States over the last decade, sexual and gender minority (SGM) individuals continue to face significant health disparities, driven partly by disproportionately higher rates of self-reported discrimination and harassment when seeking healthcare. Historically, physicians have received little to no required training on how to provide sensitive, competent care to SGM patients, and continue to demonstrate poor competency with SGM topics despite calls for increased education and published guidelines to promote competency. The present study aimed to investigate competency with SGM topics among both faculty and medical students at one institution.

**Methods:**

The authors distributed an anonymous online survey (2020–2021) to medical students and student-facing faculty at one allopathic medical school in the United States. The objective of the study was to evaluate knowledge, clinical skills, and self-reported competence with SGM topics.

**Results:**

Of survey respondents, 223 medical students and 111 faculty were included in final analysis. On average, medical students were significantly more likely to answer General Knowledge questions correctly (97.2%) compared to faculty (89.9%). There were no significant differences in responses to Clinical Knowledge questions between medical students and faculty. however medical students were significantly more likely to report competence with eliciting a thorough sexual history, and faculty were significantly more likely to report receiving adequate clinical training and supervision to work with lesbian, gay, and bisexual patients.

**Conclusions:**

Medical students demonstrated significantly higher general knowledge about SGM topics compared to faculty. Medical students and faculty demonstrated similarly low average clinical knowledge, with percent correct 65.6% for students and 62.7% for faculty. Despite significant differences in general knowledge and low clinical knowledge, medical students and faculty self-reported similar levels of competence with these topics. This indicates insufficient curricular preparation to achieve the AAMC competencies necessary to care for SGM patients.

**Supplementary Information:**

The online version contains supplementary material available at 10.1186/s12909-023-04849-2.

## Background

Sexual and gender minority (SGM) individuals make up 3–12% of the United States population [1, 2]. In 2014, the American Association of Medical Colleges’ [3]. Advisory Committee on Sexual Orientation, Gender Identity and Sex Development (AXIS) published a list of core competencies for medical schools to direct their coverage of SGM topics. However, despite calls to develop workforce competency around SGM-related issues, disparities in health outcomes for SGM individuals persist [3, 4]. SGM individuals experience disproportionate rates of psychiatric disorders, substance use, and suicide, [5–7] face higher rates of cancer, experience disparities across the cancer care continuum, [6] and miss opportunities for screening for cancer and sexually transmitted infections [6, 8].

Disparities in SGM health outcomes are due in part to inadequate training [9–12] and thus inadequate competence of physicians in SGM health topics [3, 13–15]. The competence of healthcare providers to provide care to SGM patients has been studied, with healthcare providers self-reporting competence ranging from 19.8–94% [10, 14, 16–20] and assessments demonstrating competence ranging from 24–67% [17, 20–23]. Several studies found that physicians do not routinely assess sexual history during general patient encounters, [19] in part due to fear of offending their patients, [24] and as a result, questions related to gender identity, sexual orientation, and sexual behavior are often left unaddressed [19, 25] Transgender patients in particular report that the largest barrier to healthcare access is a shortage of healthcare providers who are knowledgeable about transgender medicine [26, 27]. In addition to lack of preparation to care for SGM patients, [22, 28, 29] many physicians also exhibit beliefs and behaviors that stigmatize SGM individuals [30, 31]. One study of emergency medicine resident physicians found that 4.7% did not believe that LGBT patients deserved the same level of quality care as other patients [19]. Gender, race, religious background, language, and ethnicity also influence healthcare providers’ attitudes towards SGM people [30, 32]. SGM individuals report experiencing disproportionately high rates of discrimination when seeking healthcare. Reported negative experiences include healthcare providers being physically rough or abusive, use of excessive precautions, and refusal to provide needed care [15, 33]. These gaps in both clinical and cultural competence have been shown to impact healthcare use by SGM individuals and may worsen the health disparities they face [34].

Physician competence in SGM healthcare begins with training to care for SGM patients in medical school. Studies show that medical students are underprepared to address the needs of their SGM patients [35]: in one study, 67% of US and Canadian medical students ranked their SGM curriculum as “fair or worse” and most did not feel comfortable discussing gender-affirming care [36]. In a national survey of medical students, while 94.2% believed that SGM-based curriculum should be included in their medical education, only 31.1% of students reported that an identified SGM-based curriculum existed at their institution [37] 33% of medical schools fail to cover any SGM content during clinical years and 7% of schools lack any SGM training during pre-clinical years [38]. Together, these studies reveal the need for curricular reform and the expansion of SGM healthcare training within medical education.

Efforts to improve medical curricula on SGM topics need also to address faculty competence related to SGM healthcare. No published studies of which we are aware examine perceived competence in SGM healthcare among both medical students and faculty members.

## Methods

In this research study, we sought to examine whether faculty and medical students differ in their competence with SGM medical education topics. We use the term SGM to encompass sexual minorities and gender minorities and include people that identify as lesbian, gay, bisexual, transgender, nonbinary, gender non-conforming, and queer with respect to their sexual or gender identity. We examined the knowledge, clinical skills, and perceived competence with SGM topics among medical students and student-facing faculty at an M.D.- granting medical school in the United States, Tufts University School of Medicine (TUSM). The Tufts University Health Sciences and Social and Behavioral Research Institutional Review Board reviewed this study and classified it as exempt with informed consent.

### Survey design

We developed a 35-question online Qualtrics survey, adapting survey items from the AAMC core SGM competencies and previously published SGM self-assessment tools [3, 17, 21, 37, 39, 40]. To assess face and expert validity, we designed these questions in collaboration with medical school community members, SGM-identified community members, and SGM health experts. We then piloted the survey with two physician assistant (PA) students at TUSM and two TUSM faculty members.

For questions specifying populations within the SGM community, we used “sexual minorities” and “gender minorities.” We defined “sexual minorities” as “individuals that engage in non-heterosexual sexual behaviors and that may identify as lesbian, gay, bisexual, queer, or pansexual.” We defined “gender minorities” as “individuals whose gender or sex assigned at birth is discordant with their chosen gender identity or gender expression. Gender minorities include individuals that are transgender, gender queer, non-binary, or that are born with disorders of sex development.”

We collected demographic information as a priori. Respondents were invited to select all outcomes that applied to their identity. Respondents were also able to select “Other” for gender identity (*n* = 1), sexual orientation (*n* = 0), and race/ethnicity (*n* = 1), and type-in responses were analyzed individually for categorization. Respondents who self-identified as “medical student” included students who at the time of survey completion had completed one (*n* = 66), two (*n* = 53), three (*n* = 57), or four (*n* = 22) years of medical school curriculum at this institution. This also included students who at the time of survey completion were doing research as part of an MD/PhD program (*n* = 0), doing a research year as part of the MD or combined MD program (*n* = 9), and those who were on a leave of absence (*n* = 0). Respondents who self-identified as “faculty” included those who identified themselves as “lecturing graduate student,” “clerkship director,” “lecturer,” “course director,” “program director,” or “preceptor.”

### Recruitment

The 35-question web-based survey was available from July 1, 2020 through February 10, 2021 and open to medical students and faculty who self-identified as student-facing. We administered the survey using Qualtrics (Provo, UT, USA) in compliance with Institutional Review Board regulations and policies, and obtained informed consent prior to participants beginning the survey.

We recruited participants with convenience sampling. We distributed email invitations through the medical school’s Office of Educational Affairs, the Office of Student Affairs, and the medical school student government officers (see Additional file 5). Additionally, we distributed invitations through Facebook posts on closed invite-only TUSM medical student pages. To limit sampling bias, we did not approach the SGM medical student organization at TUSM to distribute the survey. We did not provide potential participants with compensation or incentives to respond to the survey.

### Analytic strategy

We compared responses from medical students (*n* = 223) and faculty (*n* = 111) using chi-square tests and two proportion *t* tests, where appropriate. We performed statistical tests using Stata/SE version 16.1 for Windows (Stata Corp LLC, College Station, TX). We also assessed knowledge on an aggregated level by calculating the mean number of correct answers across the 5 General Knowledge questions, and across the 4 Clinical Knowledge questions. Clinical domain questions were further analyzed with subpopulations of pre-clinical (*n* = 119) vs. clinical (*n* = 88) medical students and non-clinical (*n* = 22) vs. clinical faculty (*n* = 64). To compare self-reported competence between medical students and faculty, we conducted Mann-Whitney U Tests to preserve the ordinal nature of the response variables (from strongly agree to strongly disagree).

For applicable General Knowledge questions, we determined that applicable question responses of both “False” and “Can’t Say” were correct. For domains using Likert scales, we excluded participant responses of “I don’t know” from analysis.

We dropped Question 10 from analysis and aggregated Clinical Knowledge analysis due to question wording leading to ambiguity in analysis of the outcomes. Thus, the apparent % correct could be falsely elevated and mask the rate of misinformation on this topic.

## Results

### Respondent characteristics

A total of 428 individuals initiated surveys. We excluded any survey where more than 20% of the answers were incomplete (*n =* 88). In addition, we excluded individuals declining to identify their affiliation with the medical school (*n* = 2) and those who designated their affiliation as “Other” (*n* = 4). This left 334 responses for analysis (Table 1), including 223 medical students and 111 faculty, with an estimated response rate of 27.2% (223/820) for medical students and 2.8% (111/4,000) for faculty.

**Table 1:** Demographic characteristics of medical student and faculty respondents (n = 334) to an online survey of participants at one institution (Boston, MA) about competence with SGM content, 2020–2021

Role at TUSM		Medical Students (n = 223)	Faculty (n = 111)	P value^a^ (significant differences bold)
Age		**223**	**111**	**P < .001**
	21–30	211 (94.6%)	0	
	31–40	11 (4.9%)	29 (26.1%)	
	41–50	0	32 (28.8%)	
	51–60	0	27 (24.3%)	
	61–70	0	13 (11.7%)	
	71–80	0	5 (4.5%)	
	81+	0	4 (3.6%)	
	Prefer not to answer	1 (0.4%)	1 (0.9%)	
Gender identity^b^		**222**	**109**	P = .07
	Woman or female	141 (63.5%)	55 (50.5%)	
	Man or male	76 (34.2%)	51 (46.8%)	
	Trans or gender-diverse^c^	5 (2.3%)	3 (2.8%)	
Sexual orientation^b^		**224**	**109**	P = .18
	Heterosexual	176 (78.6%)	93 (85.3%)	
	Lesbian, gay, bisexual, queer, pansexual, or asexual	43 (19.2%)	16 (14.7%)	
	I’m not sure	5 (2.2%)	0	
Race/ethnicity^b^		**257**	**112**	P = .12
	White	176 (68.5%)	88 (78.6%)	
	Black or African American	8 (3.1%)	1 (0.9%)	
	Latinx or Hispanic	11 (4.3%)	0	
	Asian/ Asian American Pacific Islander	42 (16.3%)	15 (13.4%)	
	Native or Indigenous	1 (0.4%)	0	
	Arab/ Middle Eastern	9 (3.5%)	2 (1.8%)	
	Biracial or multiracial	10 (3.9%)	6 (5.4%)	

a- Fisher’s exact p-value from Chi-square test

b- Respondents were invited to select all outcomes that applied to their identity. For analysis, each identity outcome was coded as a binary yes/no response

c- Several outcome responses were combined in aggregate for analysis. “Trans or gender-diverse” encompasses respondent selections of “transgender,” “trans man,” “trans woman,” “genderqueer,” “gender non-conforming,” “non-binary,” “gender expansive,” “gender-diverse,” and “gender fluid.”

### General knowledge

On average, medical students were significantly more likely to answer General Knowledge questions correctly compared to faculty (97.2% vs. 89.9%, p = .03 from two-sided t-test, Table 2). When analyzed question by question, medical students were significantly more likely to correctly answer three of five General Knowledge questions. These questions served as proxies for adequate knowledge based on AAMC competency 1^3^: adequate knowledge about differences between gender expression and gender identity (Question 3), differences between sex and gender (Question 4), and differences between sexual orientation and sexual behavior (Question 5).

Medical students were significantly more likely compared to faculty to correctly identify that a patient’s pronouns cannot be determined based on gender expression and chosen name (Question 3; *n* = 221 (99.1%) vs. *n* = 104 (93.7%), P < .007 Fisher’s exact from Chi-square test). Medical students were also significantly more likely compared to faculty to correctly identify that a patient’s gender cannot be determined based on sex assigned at birth and pronouns (Question 4; *n* = 217 (97.7%) vs. *n* = 94 (84.7%), P < .001 Fisher’s exact from Chi-square test). Finally, medical students were significantly more likely to correctly identify that sexual orientation cannot be determined from sexual behavior (Question 5; *n* = 211 (94.6%) vs. *n* = 92 (82.9%), P = .001 Fisher’s exact from Chi-square test).

**Table 2:** % of participants answering correctly on general knowledge questions in SGM health topics, by medical students and faculty, from respondents to an online survey at one institution (Boston, MA) about competence with SGM content, 2020–2021

	Question (Correct Answer)	Medical Students (n= )	Faculty (n= )	P value^a^ (significant differences bold)
		% answering correctly		
1	The definition of gender expression is the way in which a person expresses their gender identity, typically through their appearance, dress, or behavior. (**TRUE**)	95.5% (n = 213/223)	90.9% (n = 100/110)	P = .14
2	All men who have sex with men are gay. (**FALSE** or **CAN’T SAY**)	99.1% (n = 221/223)	97.3% (n = 108/111)	P = .34
3	Patient presents to clinic for wellness check after establishing care at your office. On the patient’s medical record, the patient’s sex indicates that they are female. The patient introduces themselves to you as Charlie. Charlie reports that they are concerned about birth control. You offer Charlie multiple options for contraception. Charlie has masculine features and is dressed in men’s clothes. Is the following statement true or false? Charlie uses he/him/his pronouns. (**FALSE** or **CAN’T SAY**)	99.1% (n = 221/223)	93.7% (n = 104/111)	**P = .007**
4	Max introduces themself and states that they prefer to use the pronouns they/them/theirs. Max was assigned female at birth. Is the following statement true or false? Max is a woman. (**FALSE** or **CAN’T SAY**)	97.7% (n = 217/222)	84.7% (n = 94/111)	**P < .001**
5	Leah is a 32-year old woman that works at a grocery store as a manager. Leah goes to her doctor for a wellness check. The doctor asks Leah a series of questions about her sexual history. Leah reports that she has never been pregnant, has had sex in the past with both men and women, and that she is currently sexually active with one woman. Is the following statement true or false? Leah identifies as a bisexual. (**FALSE** or **CAN’T SAY**)	94.6% (n = 211/223)	82.9% (n = 92/111)	**P = .001**
	Mean (Standard Deviation) number of correct answers	97.2% (1.85%)	89.9% (5.41%)	**P = .03**

a- Fisher’s exact p-value from Chi-square or t-test where appropriate

### Clinical knowledge

In aggregate and when analyzed question by question, we found no significant differences in correct responses for medical students compared to faculty members for the Clinical Knowledge questions (Table 3).

**Table 3:** % of participants answering correctly on clinical knowledge questions in SGM health topics, by medical students and faculty, from respondents to an online survey at one institution (Boston, MA) about competence with SGM content, 2020–2021

	Question (Correct Answer)	Medical Students (n= )	Faculty (n= )	P value^a^ (significant differences bold)
		% answering correctly		
6	HPV-associated cervical dysplasia can be found in lesbians with no history of heterosexual intercourse. (**TRUE**)	76.2% (n = 170/223)	78.4% (n = 87/111)	P = .68
7	Research indicates that individuals that identify as lesbian, gay, and bisexual experience lower levels of mental health conditions compared to heterosexual individuals. (**FALSE**)	80.7% (n = 180/223)	82.0% (n = 91/111)	P = .88
8	Regularly screening gay and bisexual men for anal cancer through anal Pap testing can increase life expectancy. (**TRUE**)	59.6% (n = 133/223)	54.1% (n = 60/111)	P = .35
9	Patient presents to clinic for wellness check after establishing care at a family practice. The patient presents themselves to the doctor as Charlie. Charlie identifies as a trans-man and takes gender-affirming hormones. Charlie reports that he is fearful of becoming pregnant and that he is interested in taking birth control. Charlie has no additional medical or family history that would preclude him from taking contraception. It is appropriate to offer Charlie all forms of contraception. (**TRUE**)	45.7% (n = 102/223)	36.4% (n = 40/110)	P = .13
10	Delaying puberty in children experiencing gender dysphoria using puberty-blocking treatment has no impact on odds of lifetime suicidal ideation and psychological distress compared to those who do not receive it. (**FALSE**)	*Not analyzed after team discussion due to lack of directionality in question wording leading to potential ambiguity in analysis of responses*		
	Mean (Standard Deviation) number of correct answers	65.6% (13.9%)	62.7% (18.6%)	P = .84

a-Fisher’s exact p-value from Chi-square or t-test where appropriate

In subgroup analyses of Clinical Knowledge questions for pre-clinical vs. clinical medical students, we found no significant differences in aggregate (see Additional file 1). Significant differences between pre-clinical and clinical medical students were identified for two individual questions (Question 6 and Question 7, see Additional file 1).

In subgroup analyses of Clinical Knowledge questions for pre-clinical vs. clinical faculty, we found no significant differences in aggregate (see Additional file 2). Significant differences between pre-clinical and clinical faculty were identified for one individual question out of four analyzed (Question 9, see Additional file 2).

We found no significant differences in clinical knowledge for clinical faculty vs. clinical medical students in aggregate or when analyzed question by question (see Additional file 3).

### Self-reported competence with clinical care

We evaluated self-reported competence with clinical SGM topics for medical students and faculty (Table 4). We found no significant differences in the distribution of responses for the majority, eight of ten, question domains (Table 4). We found significant differences in the distribution of responses for only two questions (Question 11 and Question 20). Medical students were significantly more likely than faculty to report competence with eliciting a thorough sexual history regardless of sexual orientation or gender identity (Question 11; *n* = 162 (73.7%) vs. *n* = 63 (58.3%), P = .02 Wilcoxon rank sum). Faculty were significantly more likely to report receiving adequate clinical training and supervision to work with lesbian, gay, and bisexual patients (Question 20; *n* = 53 (48.2%) vs. *n* = 75 (33.6%), P = .02 Wilcoxon rank sum). The significant differences in perceived competence with eliciting a thorough sexual history persisted in sub-analyses of clinical medical students compared to clinical faculty (see Additional file 4; Question 11; *n* = 64 (73.5%) vs. *n* = 37 (57.8%), P = .03 Wilcoxon rank sum).

**Table 4:** – Self-reported competence with clinical care for SGM patients, by medical students and faculty, from respondents to an online survey at one institution (Boston, MA) about competence with SGM content, 2020-2021^b^

	Question	Population	Strongly agree	Agree	Neutral	Disagree	Strongly disagree	P value^a^ (bold differences significant)	
11	I can elicit a thorough sexual history from a patient in a sensitive and effective manner regardless of their sexual orientation or gender identity.	Medical Students (n = 220)	16.4%	57.3%	17.7%	6.8%	1.8%	**P = .02**	
		Faculty (n = 108)	12.0%	46.3%	32.4%	6.5%	2.8%		
12	I ask every new patient their preferred pronouns, or if a patient volunteers their preferred pronouns, I confirm this information with the patient’s chart.	Medical Students (n = 223)	8.1%	27.4%	22.9%	30.9%	8.1%	P = .61	
		Faculty (n = 110)	3.6%	17.3%	21.8%	44.5%	10%		
13	I feel competent managing the care of a transgender patient on gender-affirming hormone replacement therapy in a developmentally appropriate manner.	Medical Students (n = 223)	2.7%	13.5%	15.7%	38.6%	23.3%	P > .99	
		Faculty (n = 108)	2.8%	16.7%	16.7%	36.1%	25.9%		
14	I feel competent discussing safe sex practices with patients that identify as lesbian, gay, bisexual, or pansexual.	Medical Students (n = 223)	22.9%	39.9%	16.1%	15.7%	4.9%	P = .54	
		Faculty (n = 108)	13.9%	44.4%	14.8%	14.8%	10.2%		
15	I feel competent recognizing the unique health risks and challenges of sexual and gender minority individuals.	Medical Students (n = 223)	8.1%	44.4%	21.5%	21.1%	4.5%	P = .99	
		Faculty (n = 108)	3.7%	43.5%	20.4%	24.1%	8.3%		
16	When caring for LGBTQ individuals, I feel competent screening for and addressing trauma, substance use, mental health conditions, and high-risk behaviors.	Medical Students (n = 223)	11.2%	40.8%	25.6%	16.1%	4.5%	P = .77	
		Faculty (n = 108)	16.7%	39.8%	16.7%	20.4%	5.6%		
17	I feel competent defining and distinguishing the following terms: sex, gender, gender expression, gender identity, gender discordance, gender nonconformity, and gender dysphoria.	Medical Students (n = 223)	22.0%	56.5%	11.7%	8.5%	1.3%	P = .20	
		Faculty (n = 110)	11.8%	40.0%	16.4%	28.2%	3.6%		
18	I feel competent defining and describing the differences between sexual orientation, sexual identity, and sexual behavior.	Medical Students (n = 223)	32.7%	53.8%	7.6%	4.9%	0.9%	P = .75	
		Faculty (n = 110)	20.9%	41.8%	16.4%	19.1%	0.9%		
19	I have received adequate clinical training and supervision to work with transgender patients.	Medical Students (n = 223)	1.3%	7.2%	17.5%	41.7%	29.1%	P = .22	
		Faculty (n = 110)	3.6%	10.0%	19.1%	44.5%	21.8%		
20	I have received adequate clinical training and supervision to work with lesbian, gay, and bisexual patients.	Medical Students (n = 223)	6.7%	26.9%	28.7%	24.7%	9.9%	**P = .02**	
		Faculty (n = 110)	7.3%	40.9%	21.8%	22.7%	6.4%		

a-P-value from Wilcoxon rank sum test

b-Respondents who selected “I don’t know” were excluded from the analysis

## Discussion

It has been well-established that SGM patients face healthcare disparities and that this is due in part to inadequate medical education training and cultural competence of physicians regarding SGM health topics [3, 13, 35]. Our current study builds upon this growing body of knowledge by highlighting knowledge gaps between students and the faculty that teach them. Our data show that medical students demonstrate significantly higher general knowledge of SGM topics than their faculty. While medical students significantly outperformed faculty in this domain, we found that both medical students and faculty scored close to 90% correct on average, suggesting that both groups exhibited adequate mastery of general knowledge (Table 2). We found no significant differences in clinical knowledge between medical students and faculty, with a relatively low average percent correct, 65.6% (13.9% SD) and 62.7% (18.6% SD) for students and faculty, respectively (Table 3). Thus, while there was not a significant difference between students and faculty performance, neither group performed well. Clinical competence with these topics is recommended by the AAMC [3]. These results suggest that both faculty and students do not exhibit the clinical knowledge recommended by the AAMC, underscoring the need to not only improve medication education, but to require continuing education related to SGM topics for faculty.

While medical students and faculty demonstrated relatively low levels of clinical knowledge, they both perceived their clinical competence with these topics similarly (Table 4). This finding emphasizes the importance of using knowledge-based questions to assess skills and competence, as questions assessing perceived competence may not capture true gaps in knowledge related to SGM healthcare. For example, expanding the number of basic and clinical knowledge questions in the survey may better identify knowledge gaps among faculty and students, thereby providing useful information when developing curricula focusing on SGM healthcare. Our clinical competence data show two significant differences. Students were more likely to agree and strongly agree with feeling competent in their ability to elicit a thorough and sensitive sexual history (Question 11). In contrast, faculty were statistically more likely than medical students to report that they received adequate clinical training and supervision to work with lesbian, gay, bisexual and pansexual patients (Question 20). This finding is not surprising considering that clinical faculty have undergone supervised post-graduate medical education and have additional years of experience working directly with patients. Despite the significant difference in responses to Question 20, many respondents disagreed or strongly disagreed with this statement (*n* = 77 (34.6%) of medical students, *n* = 32 (29.1%) of faculty), indicating that they did not feel adequately prepared to work with sexual minority patients. Thus, there is significant room to improve both medical student and faculty clinical training and clinical supervision around working with sexual minority patients. Importantly, both faculty and medical students indicate that they have not received adequate training and supervision to work with transgender patients (Question 19). This data is consistent with previously published data that medical students report lower levels of competence related to transgender healthcare when compared to sexual minority healthcare [39].

Our study has several strengths. It is the first study of which we are aware to compare faculty and medical student knowledge, self-perceived competence, and attitudes relating to SGM topics. Additionally, this study highlights clear deficits in clinical knowledge that can be addressed through medical education reform and continuing education initiatives for faculty.

This study has several limitations to internal validity. First, our sample likely has a response bias for respondents who have an interest in SGM health topics. We attempted to reduce response bias by recruiting solely from the general medical student and faculty population. The percentage of medical student respondents identifying as lesbian, gay, bisexual, queer, pansexual, asexual in our study (*n* = 43 (19.2%)) is higher than an AAMC-administered nonrepresentative survey of 2nd year medical students, who reported that 5.9% identify as lesbian, gay, or bisexual [41, 42]. However, a respondent’s identity as part of the SGM community does not necessarily result in greater perceived competence with SGM topics in medical education. The low estimated response rate of 2.8% for faculty may be due in part to response bias, although the total number of faculty (4,000) is much greater than the number of faculty who interact directly with medical students, so this response rate is likely an underestimate. Second, age may play a confounding role in knowledge about and attitudes towards SGM individuals as there was a significant difference in the distribution of age between medical students and faculty (Table 1; P < .001). Third, social desirability bias is likely present, with respondents self-reporting greater comfort with or preparedness to care for SGM patients. For example, both medical students and faculty self-reported high competence as it pertains to SGM-inclusive healthcare, although they scored poorly on the clinical knowledge questions. Fourth, it is possible that respondents were misclassified, given our reliance on their self-reported affiliated with TUSM.

This work has several limitations to external validity. First, the survey was conducted at only one institution located in the United States which, compared to the aggregated medical student population of U.S. medical schools, is slightly enriched for students that identify as “White” (51.7% vs. 48.5%) [41, 42] s, we used convenience sampling, so we must consider the contribution of selection bias. For example, people who identify as part of the SGM community and their allies may be more likely to take the survey. We also investigated the potential for differential response to the survey based on faculty age. However, the average age of current full-time faculty at all U.S. medical schools is 48.6 years, [43] which corresponds with our faculty survey respondents, wherein the majority of faculty were in their fourth decade of life (Table 1). Future research should address these limitations. In addition, we recommend updating survey outputs for clarity of analysis, including making output selection for true/false questions binary. We would also update survey questions to improve question ambiguity. Finally, we would distribute the survey at more than one institution to improve generalizability of the results.

## Conclusions

Our data demonstrate that knowledge gaps regarding SGM healthcare exist between faculty and medical students, and that neither group demonstrates adequate clinical knowledge. This finding underscores the need for SGM healthcare education reform at the level of medical education and demonstrates that faculty also require continuing education as it relates to SGM healthcare. Despite faculty seniority, experience, and training, these data demonstrate that they are not necessarily equipped to recognize SGM healthcare disparities and that in order to improve SGM healthcare outcomes, tailoring and requiring education for both medical students and faculty is essential.

### Electronic supplementary material

Below is the link to the electronic supplementary material.

Supplementary Material 1

Supplementary Material 2

Supplementary Material 3

Supplementary Material 4

Supplementary Material 5

## Data Availability

The datasets used and/or analysed during the current study are available from the corresponding author on reasonable request.

## Declarations

### Authors’ information

AR is a psychiatry resident in the Department of Psychiatry at Tufts Medical Center. At the time of data collection, AR was a fourth year medical student at Tufts University School of Medicine. ZB is a pathology resident in the Department of Pathology and Laboratory Medicine at University of California – San Francisco. At the time of data collection, ZB was a fourth year medical student at Tufts University School of Medicine.

## Abbreviations

SGM: sexual and gender minority
AAMC: American Association of Medical Colleges
AXIS: Advisory Committee on Sexual Orientation, Gender Identity and Sex Development
TUSM: Tufts University School of Medicine
PA: physician assistant
US: United States
MD: medical doctor
