# Supplementary material for: Knowledge and perceived competence with sexual and gender minority healthcare topics among medical students and medical school faculty
Source: BMC Med Educ. 2023 Dec 8;23:928. doi: 10.1186/s12909-023-04849-2 (PMC10709858; doi:10.1186/s12909-023-04849-2)
Supplement: Supplementary file 1 — Supplementary Material 1 [file 12909_2023_4849_MOESM1_ESM.docx]

**Additional file 1** – % of participants answering correctly on clinical knowledge questions in SGM health topics, by pre-clinical medical students and clinical medical students, from respondents to an online survey at one institution (Boston, MA) about competence with SGM content, 2020-2021

|  | *Clinical Knowledge* | Pre-clinical medical students | Clinical medical students | P value^a^ **(significant differences bold)** |
| --- | --- | --- | --- | --- |
| 6 | HPV-associated cervical dysplasia can be found in lesbians with no history of heterosexual intercourse. (**TRUE**) | 68.9%  (n=82/119) | 87.5%  (n=77/88) | **P = .002** |
| 7 | Research indicates that individuals that identify as lesbian, gay, and bisexual experience lower levels of mental health conditions compared to heterosexual individuals. (**FALSE**) | 74.8%  (n=89/119) | 90.9%  (n=80/88) | **P = .003** |
| 8 | Regularly screening gay and bisexual men for anal cancer through anal Pap testing can increase life expectancy. (**TRUE**) | 65.5%  (n=78/119) | 53.4%  (n=47/88) | P = .09 |
| 9 | Patient presents to clinic for wellness check after establishing care at a family practice. The patient presents themselves to the doctor as Charlie. Charlie identifies as a trans-man and takes gender-affirming hormones. Charlie reports that he is fearful of becoming pregnant and that he is interested in taking birth control. Charlie has no additional medical or family history that would preclude him from taking contraception. It is appropriate to offer Charlie all forms of contraception. (**TRUE**) | 47.1%  (n=56/119) | 46.5%  (n=41/88) | P > .99 |
|  | Mean (Standard Deviation) number of correct answers | 64.1% (10.3%) | 69.6% (19.8%) | P = .68 |

a-Fisher’s exact p-value from Chi-square or t-test where appropriate
