# Supplementary material for: Knowledge and perceived competence with sexual and gender minority healthcare topics among medical students and medical school faculty
Source: BMC Med Educ. 2023 Dec 8;23:928. doi: 10.1186/s12909-023-04849-2 (PMC10709858; doi:10.1186/s12909-023-04849-2)
Supplement: Supplementary file 4 — Supplementary Material 4 [file 12909_2023_4849_MOESM4_ESM.docx]

**Additional file 4** – Self-reported competence with clinical care for SGM patients, by clinical medical students and clinical faculty, from respondents to an online survey at one institution (Boston, MA) about competence with SGM content, 2020-2021^b^

|  | Question | Population | Strongly  agree | Agree | Neutral | Disagree | Strongly disagree | P value^a^ **(bold differences significant)** |
| --- | --- | --- | --- | --- | --- | --- | --- | --- |
| 11 | I can elicit a thorough sexual history from a patient in a sensitive and effective manner regardless of their sexual orientation or gender identity. | Clinical Medical Students  (n=87) | 19.5% | 54.0% | 19.5% | 6.9% | 0.0% | **P = .03** |
|  |  | Clinical Faculty  (n=64) | 9.4% | 48.4% | 34.4% | 6.3% | 1.6% |  |
|  | | | | | | | | |
| 12 | I ask every new patient their preferred pronouns, or if a patient volunteers their preferred pronouns, I confirm this information with the patient's chart. | Clinical Medical Students  (n=87) | 2.3% | 16.1% | 27.6% | 46.0% | 8.0% | P = .79 |
|  |  | Clinical Faculty  (n=64) | 1.6% | 15.6% | 23.4% | 50.0% | 9.4% |  |
|  | | | | | | | | |
| 13 | I feel competent managing the care of a transgender patient on gender-affirming hormone replacement therapy in a developmentally appropriate manner. | Clinical Medical Students  (n=88) | 1.1% | 13.6% | 19.3% | 43.2% | 22.7% | P = .24 |
|  |  | Clinical Faculty  (n=64) | 1.6% | 12.5% | 20.3% | 34.4% | 31.3% |  |
|  | | | | | | | | |
| 14 | I feel competent discussing safe sex practices with patients that identify as lesbian, gay, bisexual, or pansexual. | Clinical Medical Students  (n=88) | 27.3% | 37.5% | 15.9% | 17.0% | 2.3% | P = .32 |
|  |  | Clinical Faculty  (n=64) | 14.1% | 50.0% | 17.2% | 9.4% | 9.4% |  |
|  | | | | | | | | |
| 15 | I feel competent recognizing the unique health risks and challenges of sexual and gender minority individuals. | Clinical Medical Students  (n=88) | 8.0% | 43.2% | 23.9% | 23.9% | 1.1% | P = .60 |
|  |  | Clinical Faculty (n=64) | 3.1% | 45.3% | 15.6% | 29.7% | 6.3% |  |
|  | | | | | | | | |
| 16 | When caring for LGBTQ individuals, I feel competent screening for and addressing trauma, substance use, mental health conditions, and high risk behaviors. | Clinical Medical Students  (n=87) | 13.8% | 39.1% | 28.7% | 17.2% | 1.1% | P = .77 |
|  |  | Clinical Faculty  (n=64) | 17.2% | 37.5% | 18.8% | 21.9% | 4.7% |  |
|  | | | | | | | | |
| 17 | I feel competent defining and distinguishing the following terms: sex, gender, gender expression, gender identity, gender discordance, gender nonconformity, and gender dysphoria. | Clinical Medical Students (n=88) | 22.7% | 62.5% | 10.2% | 4.5% | 0.0% | P = .053 |
|  |  | Clinical Faculty (n=64) | 10.9% | 35.9% | 20.3% | 28.1% | 4.7% |  |
|  | | | | | | | | |
| 18 | I feel competent defining and describing the differences between sexual orientation, sexual identity, and sexual behavior. | Clinical Medical Students (n=88) | 28.4% | 60.2% | 8.0% | 3.4% | 0.0% | P = .25 |
|  |  | Clinical Faculty (n=63) | 19.0% | 41.3% | 15.9% | 22.2% | 1.6% |  |
|  | | | | | | | | |
| 19 | I have received adequate clinical training and supervision to work with transgender patients. | Clinical Medical Students (n=88) | 0.0% | 5.7% | 19.3% | 39.8% | 35.2% | P = .11 |
|  |  | Clinical Faculty (n=64) | 1.6% | 9.4% | 18.8% | 46.9% | 23.4% |  |
|  | | | | | | | | |
| 20 | I have received adequate clinical training and supervision to work with lesbian, gay, and bisexual patients. | Clinical Medical Students (n=88) | 8.0% | 23.9% | 27.3% | 33.0% | 8.0% | P = .07 |
|  |  | Clinical Faculty (n=64) | 7.8% | 42.2% | 20.3% | 23.4% | 6.3% |  |

a-P-value from Wilcoxon rank sum test

b-Respondents who selected “I don’t know” were excluded from the analysis
