# Supplementary material for: Knowledge and perceived competence with sexual and gender minority healthcare topics among medical students and medical school faculty
Source: BMC Med Educ. 2023 Dec 8;23:928. doi: 10.1186/s12909-023-04849-2 (PMC10709858; doi:10.1186/s12909-023-04849-2)
Supplement: Supplementary file 5 — Supplementary Material 5 [file 12909_2023_4849_MOESM5_ESM.docx]

**Additional file 5** – Email invitation used to recruit medical students and faculty for an online survey at one institution (Boston, MA) about competence with SGM content, 2020-2021

You are being invited to take part in a research study being conducted by Dr. Joshua St. Louis from Tufts University School of Medicine (TUSM) because you are involved in medical education at TUSM.

If you choose to be in the study, you will be invited to complete a survey. This survey will help us learn more about the status of medical education regarding sexual and gender minority individuals. The survey will take you about 15 minutes.

Your participation in this study is completely voluntary and will not affect your standing as a student or employee at Tufts University School of Medicine. You are free to decline to participate, to stop taking the survey at any time, or to refuse to answer any individual question. You can skip questions that you do not want to answer or stop the survey at any time. There is a risk of loss of confidentiality. However, as the survey is anonymous, no one will be able to link your answers back to you. Please do not include your name or other information that could be used to identify you in the survey responses.

There are no direct benefits to you in participating in this study. You will not be compensated for your participation, and there is no cost to you to participate in this study. This study has been reviewed by the Tufts Health Sciences IRB.

Please go to the link below to complete the survey: [https://tufts.qualtrics.com/***](https://tufts.qualtrics.com/***%20%20%20jfe/form/SV_d1qWsVumFw3Q4x7)

Questions? Please contact Dr. Joshua St. Louis at [***@tufts.edu](mailto:***@tufts.edu) , Allison Rhodes at [***@tufts.edu](mailto:***@tufts.edu) , or Zachary Barbati at [***@tufts.edu](mailto:***@tufts.edu) .

Best regards,

Joshua St. Louis, MD, MPH, AAHIVS

Assistant Professor

Tufts University School of Medicine

145 Harrison Ave, Boston, MA 02111
